# Ileal Lipoma as a Leading Point of Ileocolic Intussusception in Adult Patient: Ultrasonography and CT Evaluation

**DOI:** 10.7759/cureus.26019

**Published:** 2022-06-16

**Authors:** Pratik J Bhansali, Suresh V Phatak, Bhavik S Unadkat, Prasanthi R Ghanta

**Affiliations:** 1 Radiodiagnosis, Datta Meghe Institute of Medical Sciences, Wardha, IND

**Keywords:** ct, intussusception, ileocolic, ultrasonography, lipoma

## Abstract

Intussusception in adults is an unusual finding and is commonly associated with benign or malignant mass as the leading point. A preoperative diagnosis on imaging is helpful in diagnosing intussusception along with pathology causing it and aids in further management. We present a case of ileocolic intussusception with lipoma as the lead point: classic ultrasonography and CT imaging findings are described with its postoperative confirmation.

## Introduction

The telescoping of one gastrointestinal (GI) system segment into another is known as intussusception [1]. As most adult patients come with intestinal obstruction in an emergency, the diagnosis is by laparotomy. Adult intussusception is an unusual condition that affects 1% of people who have an intestinal obstruction and accounts for 5-10% of all intussusception. Adult intussusception presents differently from children's intussusception. Ninety percent of intussusception occurrences in children are idiopathic, but 70% to 90% of cases in adults are linked to some underlying disease, of which 65% are due to benign or malignant causes [2].

## Case presentation

**Figure 1:**
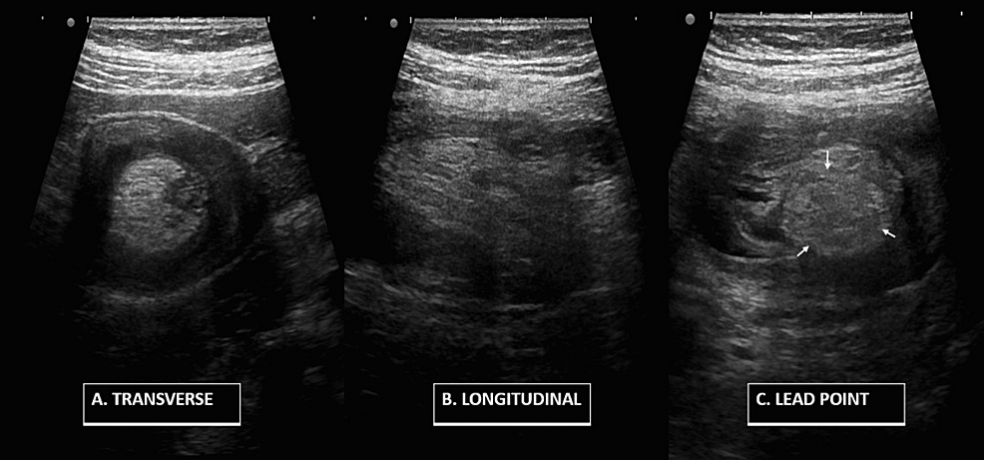
(A) USG transverse section shows bowel within bowel pattern (concentric rings of hyperechoic and hypoechoic layers), (B) USG longitudinal section shows sandwich appearance multiple parallel lines, (C) USG showing well-defined hyperechoic lipoma as a leading point (white arrow). USG: Ultrasonography

**Figure 2:**
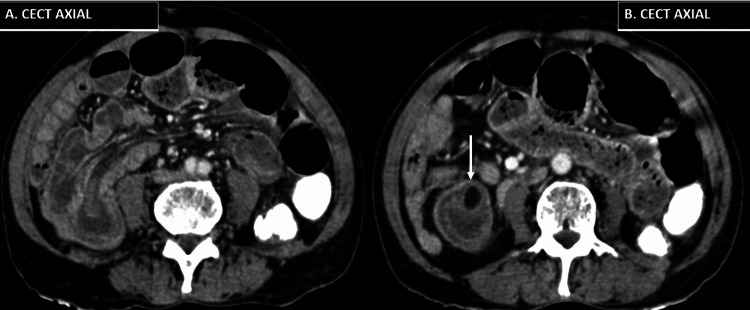
(A) Contrast-enhanced axial CT image showing bowel within bowel along with mesenteric fat and vessels, (B) Contrast-enhanced axial CT showing well-defined fat attenuation lipoma (white arrow) as the leading point of ileocolic intussusception.

**Figure 3:**
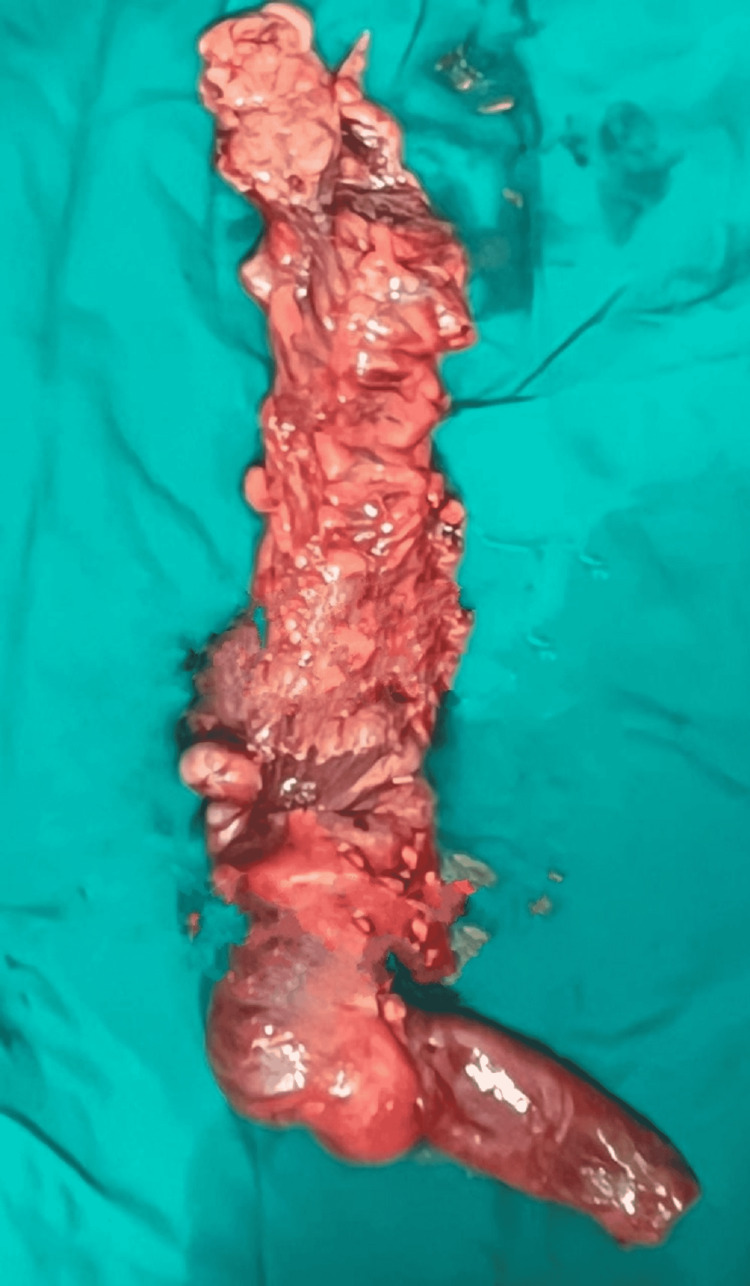
Postoperative specimen revealing irreducible ileocolic intussusception

An 80-year-old man complained of colicky abdominal pain, particularly in the right iliac region, abdominal distention, and failure to pass flatus or stools over the past three days. On physical examination, there was abdominal distension associated with mild abdominal tenderness but no discernible abdominal mass. Vital indicators such as temperature, pulse, blood pressure, and respiratory rate were within normal limits. A bowel within bowel pattern (concentric rings of hyperechoic and hypoechoic layers) (Figure 1A) was revealed by abdominal sonography. The ultrasonography (USG) revealed features of “sandwich appearance”, multiple parallel lines in the longitudinal section (Figure 1B). An attempt to trace the lead point revealed a hyperechoic lesion with linear strands within and was well-defined, suggesting it to be a lipoma (Figure 1C). Further contrast-enhanced computed tomography (CECT) abdomen demonstrated an ileocolic intussusception with lipoma as its leading point (Figure 2A, 2B). Laparotomy was performed for the patient, and a non-reducible ileocolic intussusception was located. The lead point was tracked down and came out to be a well-defined mass lesion (Figure 3). Resection of both ends was done, followed by end-to-end anastomosis between the ileum and colon. The post-operative period was uneventful, and symptoms were relieved. The histopathological report of the sample confirmed it to be a lipoma. The patient is doing well on follow-up.

## Discussion

Intussusception is uncommon in adults with difficult clinical diagnoses due to the lack of specific symptoms. Mechanism of intussusception in adults is considered to happen when the peristaltic motion of the bowel drags an intraluminal mass forward, along with the connected intestinal tract with it. The typical examples of this category include pedunculated tumours, such as lipomas or adenomatous polyps [3,4]. Early detection of intussusception can help to avoid intestinal necrosis, and in certain situations, it can be life-saving [5]. Considering nonspecific symptoms, diagnostic imaging is crucial in making a diagnosis. USG and CT are the most regularly utilised diagnostic modality for diagnosis. Acute intussusception must be diagnosed as soon as possible since it causes intestinal obstruction and gut ischaemia [6].

As it is both inexpensive and readily available, non-invasive ultrasonography is usually the first investigation in suspected intussusception. Two typical characteristics, “target and doughnut sign”, are seen on the transverse section on USG, and the longitudinal view demonstrates a “pseudo kidney sign”. Ultrasonography has a diagnostic accuracy of 78.5% and 86.6% in cases of palpable abdominal mass before surgery [7].

Although ultrasonography has higher accuracy in children, adults require computed tomography (CT) to make a diagnosis, as an underlying pathology needs to be evaluated and to locate a lead point. Three distinct patterns of intussusception have been identified at CT. A target shape is appreciated on CT when the long axis of the intussusception is at a right angle to the beam. When the longitudinal axis of the intussusception is in line with the beam, it is seen as a sausage-shaped mass lesion which shows an alternating intestinal wall and mesenteric fat appearing in high and low attenuation bands. Once oedema, vascular compromise and mural thickening sets in, leading to the visualisation of a reniform lump which gives a pseudo kidney appearance on imaging [8]. Once the pressure within the bowel elevates, venous flow, followed by arterial flow, becomes obstructed. An early diagnosis becomes critical to prevent dreaded complications like bowel ischemia and perforation [9].

Even though lipomas are unusual in the GIT system, they can develop in the large intestine in up to 70% of cases or in the small intestine in 30% of cases. Lipomas of the small intestine are generally solitary, although they can sometimes be numerous, more commonly located in the ileum, jejunum, followed by duodenum [10]. Ninety to 95% of lipomas are submucosal or subserosal in location [11]. Submucosal lipomas commonly function as a lead point in the intussusceptum [10].

Although this case report presents adult intussusception with lipoma as a leading point, most of the literature suggests malignant etiologies like adenocarcinoma, leiomyosarcoma, lymphoma and metastatic mass lesions as a lead point in adult intussusception [12].

## Conclusions

Adult intussusception is uncommon and usually associated with benign or malignant tumours. USG and CT are sensitive and specific in the early diagnosis of intussusception and also in detecting the underlying pathology leading to intussusception. An accurate and timely diagnosis as in our case helps in better management and reduces the chances of complications.
